# The Body Fluids in Cancer

**Published:** 1910-03-19

**Authors:** 


### The Body Fluids in Cancer.

In the *New York Medical Record* of February 26 Dr. Eugene Hodenpyl, pathologist to the Roosevelt Hospital, gives a concise and modest account of some experimental work on the treatment of cancer, especially inoperable cases, which we commend heartily to the consideration of everyone interested in the subject of cancer and its cure. The present tendency in cancer research is, we believe unfortunately, in favour of laboratory experiments, animal studies, and tests *in vitro* rather than towards clinical investigation. While it is undoubtedly true that much may be learned from a study of the comparative pathology of cancer, it is regrettable that investigators have not been more active in following up certain interesting facts already elucidated and well known to clinicians. How fruitfully interesting such a pursuit can be will be readily seen by anyone who takes the trouble to glance through Dr. Hodenpyl's brief paper, which we have no hesitation in saying is one of the most important contributions to the literature of inoperable carcinoma that has yet appeared.

Four years ago Dr. Hodenpyl became interested in an inoperable, hopeless case of recurrent carcinoma of the breast in a woman of 37, in which secondary deposits in the liver and elsewhere, with ascites, could be demonstrated. To his great surprise the woman practically recovered. "The abdominal tumours gradually grew smaller and became imperceptible, while the liver became smoother and smaller. With the exception of the scars and extreme chyliform ascites, requiring frequent tapping, there is now no indication of the original disorder." In speculating upon this unexpected termination, the author "was led to weigh the possibility of the development by the patient of some sort of antibody inimical to the progressive growth and persistence of the tumour cells. The alternative hypothesis, which seemed plausible, was that in the processes of tumour-tissue formation in the abdomen, some physical or physiological disturbance of organic or internal secretions might have occurred, leading to the accumulation or formation of substances antagonistic to tumour-cell growth or existence."

Experimenting with the ascitic fluid from this patient, he found that his results were both surprising and encouraging. Injections were made in small quantities, near or directly into the tumours, or in large quantities into the veins in selected cases of inoperable carcinoma. The general effects of these injections in man has been nearly uniformly to induce a temporary local redness, tenderness, and swelling about the tumours, which soon subside. Then occur softening and necrosis of the tumour tissue, which is now absorbed or discharged externally, with the subsequent formation of more or less connective tissue. "In all cases," the author writes, "the tumours have grown smaller; in some they have disappeared altogether. In no instance has any tissue in the body, other than the tumour, shown the least reaction after the injections. The greater number of the forty-seven cases thus far treated were distinctly unfavourable, many of them hopeless."

These results are well worth further investigation. Dr. Hodenpyl promises a complete summary of cases, with full details of his method and technique, in a future number, which will be anxiously awaited by all interested in the subject of cancer. There is a praiseworthy modesty and fairness, an absence of intimidating dogmatism, in Dr. Hodenpyl's paper which must appeal forcibly to those who have had to wade through lengthy and involved papers on cancer research, subjects in which a minimum of grain was overloaded with the maximum of chaff, while sometimes the wheat was totally absent. We shall look forward with interest to any further communications he may have to make on the subject, and we hope that clinicians in this country will not hesitate to weigh his results with a view to giving patients who are beyond benefit of surgery the chance that his method of treatment appears to hold out.

